# Thiothymidine combined with UVA as a potential novel therapy for bladder cancer

**DOI:** 10.1038/bjc.2011.180

**Published:** 2011-05-24

**Authors:** S W Pridgeon, R Heer, G A Taylor, D R Newell, K O'Toole, M Robinson, Y-Z Xu, P Karran, A V Boddy

**Affiliations:** 1 Northern Institute for Cancer Research, Medical School, Newcastle University, Newcastle NE2 4HH, UK; 2 Department of Histopathology, Royal Victoria Infirmary, Newcastle, UK; 3 Cancer Research UK London Research Institute, Clare Hall Laboratories, South Mimms, UK; 4 Department of Urology, Freeman Hospital, Newcastle, UK

**Keywords:** Thiothymidine, UVA, bladder cancer, raltitrexed, DNA damage

## Abstract

**Background:**

Thiothymidine (S^4^TdR) can be incorporated into DNA and sensitise cells to DNA damage and cell death following exposure to UVA light. Studies were performed to determine if the combination of S^4^TdR and UVA could be an effective treatment for bladder cancer.

**Methods:**

Uptake and incorporation of S^4^TdR was determined in rat and human bladder tumour cell lines. Measures of DNA crosslinking and apoptosis were also performed. *In vivo* activity of the combination of S^4^TdR and UVA was investigated in an orthotopic model of bladder cancer in rats.

**Results:**

Thiothymidine (200 *μ*M) replaced up to 0.63% of thymidine in rat and tumour bladder cancer cells. The combination of S^4^TdR (10–200 *μ*M) and UVA (1–5 kJ m^−2^) caused apoptosis and cell death at doses that were not toxic alone. Addition of raltitrexed (Astra Zeneca, Alderley Edge, Cheshire, UK) increased the incorporation of S^4^TdR into DNA (up to 20-fold at IC_5_) and further sensitised cells to UVA. Cytotoxic effect was associated with crosslinking of DNA, at least partially to protein. Intravenous administration of S^4^TdR, in combination with UVA delivered directly to the bladder, resulted in an antitumour effect in three of five animals treated.

**Conclusion:**

These data indicate that the combination of S^4^TdR and UVA has potential as a treatment for bladder cancer, and give some insight into the mechanism of action. Further work is necessary to optimise the delivery of the two components.

In the UK, transitional cell carcinoma (TCC) of the urinary bladder is the fifth most commonly diagnosed malignancy with 10 091 new cases reported in 2007 (CRUK Cancer Statistics, 2010).

The prevalence of this disease significantly exceeds its primary incidence as it has a high rate of recurrence ranging from 31% at 5 years for low-risk tumours to 78% at 5 years for high-risk tumours (Babjuk *et al*, 2008). Bladder cancer can be broadly classified based on two distinct molecular and histological TCC pathologies – nonmuscle invasive bladder cancer (NMIBC) and muscle invasive bladder cancer (MIBC) (Knowles, 2008). Approximately 75–85% of patients present with NMIBC (Parker *et al*, 1996), and these cancers are associated with a relatively benign course rarely affecting survival, however, surveillance and recurrence makes this an expensive disease to manage. In NMIBC, the presence of multifocal disease, high-grade tumours or with concomitant carcinoma *in situ* is associated with the molecular biology of the aggressive variant of TCC (MIBC). These NMIBC patients have a high risk of both recurrence and disease progression into MIBC. Once there is MIBC, contemporary overall survival at 5 years following cystectomy is 50–60% (Shariat *et al*, 2003). Intravesical Bacille Calmette-Guérin (BCG) therapy is currently the favoured treatment for high-risk patients despite significant morbidity. Treatment with BCG reduces the odds of progression by 27% in patients with intermediate- and high-risk tumours (Sylvester *et al*, 2002). Patients who fail to respond to BCG require radical and morbid surgery for the best possible chance of a cure, and new approaches to reduce progression are a clinical priority. As the bladder can be accessed readily via the transurethral route, it is ideal for topical photosensitive treatments, as exemplified by the increasing use of photodynamic bladder cancer diagnostics, and this potential is further explored in a preclinical model in this paper.

4-Thiothymidine (S^4^TdR) is a pyrimidine nucleoside thio analogue. Although it has close structural similarities to thymidine (TdR), the two molecules have markedly different ultraviolet absorbance spectra. Thymidine absorbs maximally at 270 nm (UVC) while the peak absorption of S^4^TdR is in the UVA range at 340 nm.

The altered photospectrum of S^4^TdR enables it to form photochemical DNA intrastrand crosslinks (Warren *et al*, 1998) and DNA–protein crosslinks. These properties have been used to investigate the structural and functional interactions of DNA-binding proteins with synthetic oligonucleotides containing S^4^TdR (Nikiforov and Connolly, 1992). Interstrand DNA crosslinks are also formed and S^4^TdR-containing oligonucleotides can be photoligated in aqueous solution (Liu and Taylor, 1998). Importantly, these types of photochemical DNA lesion in cellular DNA are all potentially cytotoxic.

Thiothymidine incorporation into DNA and a consequent sensitisation to UVA has been demonstrated in various cultured skin cell lines (Massey *et al*, 2001; Reelfs *et al*, 2007). The cytotoxic effect is hypothesised to result partly from the formation of bulky DNA intrastrand crosslinks. Incorporation of S^4^TdR is dependent on the thymidine salvage pathway mediated by thymidine kinase (TK). This raises the possibility that inhibiting *de novo* synthesis of thymidine nucleotides might increase DNA S^4^TdR incorporation by forcing increased reliance on thymidine salvage. Raltitrexed (N-(5[3,4-dihydro-2-methyl-4-oxoquinazolin-6-ylmethyl]-N-methylamino -2-thenoyl)-L-glutamic acid/‘Tomudex’/ZD1694) is a water soluble quinazoline-based antifolate that is a specific inhibitor of thymidylate synthase (TS), a key enzyme in the *de novo* pathway (Jackson *et al*, 1983; Mitrovski *et al*, 1994). In bladder cancer cells, TS inhibition results in both an increased TK activity and upregulated expression of the nucleotide transporter protein to maintain thymidylate levels through pyrimidine salvage (Pressacco *et al*, 1995). It is hypothesised that raltitrexed might be used to increase thiothymidine uptake into cells and incorporation into DNA and subsequently to increase the sensitivity to UVA.

## Materials and methods

### Cell culture

MYU-3L (Kameyama *et al*, 1993; Kawamata *et al*, 1993), MBT2 (Soloway *et al*, 1973), RT4 (Rigby and Franks, 1970) and AY27 bladder cancer cell lines were cultured in RPMI-1640 medium supplemented with 10% foetal bovine serum, penicillin and streptomycin in a humidified atmosphere of 95% air, 5% CO_2_ at 37°C. AY-27 TCC cells were a kind gift from Ronald Moore (Professor of Surgery and Oncology, University of Alberta), and were derived from primary rat tumours established by the oral administration of FANFT.

### Determination of the S^4^TdR content of DNA

Thiothymidine was added to culture medium to achieve concentrations of 1–200 *μ*M for two cell-doubling times (Table 1).The DNA was extracted using Qiagen DNeasy and converted to deoxynucleosides by successive digestion with DNAse I (Sigma, Poole, UK), nuclease P1 and alkaline phosphatase (Roche GmbH, Mannheim, Germany). Quantification of S^4^TdR and thymidine nucleosides was carried out using a Perkin Elmer LC System (Beaconsfield, Buckinghamshire, UK) coupled to an API-4000 LC/MS/MS Triple Quadrupole Mass Spectrometer (Applied Biosystems, Warrington, UK), with Analyst software for data acquisition and analysis. Separation was performed on a Phenomenex 20 × 4.0 mm Luna 3u C_18_ Mercury column. Mobile phase consisted of 0.05 M NH_3_Ac pH 5.0/methanol at 0.2 ml per minute. Positive ESI mode was used with *m/z* 242.9/127.1 for thymidine and *m/z* 259.1/143.0 for S^4^TdR and the limits of detection were 2.5 pg and 0.25 pg, respectively. The S^4^TdR content of DNA was expressed as the percentage of DNA thymidine replaced by S^4^TdR.

### UVA exposure

Cells cultured in dishes or plates were exposed to UVA using a Philips facial Studio HB 175 (Philips, Guildford, UK). The distance between the plates and the lamp was measured to deliver a dose rate of 5 mW cm^−2^ UVA. Under these conditions, a 1 sec exposure under the lamp corresponded to 0.05 kJ m^−2^.

### Determination of growth inhibition

Cells were cultured in the presence of S^4^TdR for two cell-doubling times. Plates were then exposed to UVA as described above and were re-incubated for a further 72 h. Cell growth was determined using the Sulforhodamine B (SRB) assay (Skehan *et al*, 1990) and growth inhibition expressed as a percentage of growth compared with untreated controls.

### Comet assay

The Comet assay was performed using components of the *CometAssayKit* (Trevigen, Oxford, UK). For UVA exposure, cells were detached from the dishes and re-suspended in PBS. Cells were re-suspended at a concentration of 5 × 10^5^ cells ml^−1^ and mixed with molten *LM Agarose* (Trevigen) in a ratio of 1 : 10 and 75 *μ*l of the suspension placed on *Comet slides* (Trevigen). Electrophoresis was carried out for 20 min at 1 volt cm^−1^ at 300 mA. Slides were stained with SYBR green and viewed under a Leica TCS SP2 UV Confocal Laser Scanning Microscope. The SYBR green was excited by the 488 nm argon laser and the emission detected at 496–549 nm. Analysis of comets was performed using Komet 5 software (Andor Technology, Belfast, Northern Ireland). Fifty comets were analysed for each treatment group to give values for the Olive tail moment and comet tail length.

Crosslinking was expressed as a percentage decrease in Olive tail moment using the formula below as previously described (Hartley *et al*, 1999). 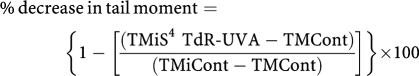 where TM iCont=tail moment of irradiated control cells; TM iS^4^TDR-UVA=tail moment of irradiated cells treated with S^4^TdR and UVA; TMCont=tail moment of unirradiated control cells.

To discriminate DNA–DNA crosslinks from DNA–protein crosslinks, a proteinase K treatment was performed. After lysis, slides were washed in TE buffer (10 mM Tris, 1 mM EDTA, pH 10), then covered with 100 *μ*l proteinase K (Sigma) (1 mg ml^−1^ TE buffer) and incubated for 2 h in a humidified incubator at 37°C. Controls were incubated with 100 *μ*l TE buffer only. Following incubation, slides were processed as above.

### Assessment of apoptosis

Caspase 3 and 7 activities were measured using the Caspase-Glo Assay (Promega, Southampton, UK). The MYU-3L and AY27 cells were incubated in the presence or absence of S^4^TdR (100 *μ*M) for two cell-doubling times and exposed to 10 kJ m^−2^ UVA. Cells were re-incubated for 24 h in fresh medium. Caspase-Glo 3/7 reagent was added and luminescence was measured.

AY27 and MYU-3L cells treated with S^4^TdR (100 *μ*M) and UVA (10 kJ m^−2^) were suspended and stained with Annexin V -FITC (Beckman Coulter, High Wycombe, UK). Samples were analysed by flow cytometry using BD FACScan II Flow cytometer (BD Biosciences, Oxford, UK).

### Orthotopic animal model

All *in vivo* work was carried out under strict regulatory approval (LREC licence PPL 60/2836). Female Fischer rats (F344) weighing 170–200 g were anaesthetised using inhaled isofluorane. Rats were catheterised using a 20-gauge cannula. The bladder was preconditioned using 0.1 N HCl to strip the epithelium and neutralised with 0.1N KOH followed by a PBS wash to create a surface suitable implantation of tumour cells. Suspensions of MYU-3L cells (1 × 10^5^ in 0.4 ml RPMI 1640 per 50% FCS) were instilled for 1 h. Rats were turned 90° every 15 min to encourage even distribution of tumour cells. Two days after tumour cell instillation, the urothelium was completely denuded, with cancer cells overlying oedematous subepithelial connective tissue. At day 5 post-instillation, 80% of tumours showed invasion into the subepithelial connective tissue consistent with stage pT1 tumours. The subendothelial connective tissue showed increased vascularity over normal rats' bladders. Tumour depth varied between 0.2–1.4 mm. By day 10, 100% of tumours progressed to muscle invasive disease (pT2). At 21 days post-instillation, all tumours were histological stage T3 with invasion into the perivesical fat and areas of tumour necrosis. The progression to invasion in the MYU-3L model without early metastases makes this a suitable model of progression in high-risk NMIBC.

### Administration of S^4^TdR and UVA light

Thiothymidine solution was dissolved in sterile water up to doses of 16 mg in 0.4 ml aliquots. An intravesical instillation time of 2 h was used. Intravenous injections of S^4^TdR (160 mg kg^−1^) were reconstituted in 1.0 ml sterile water and administered via tail vein injection. Whole bladder UVA exposure was achieved using a UVCLEAN fibre optic system (Sensor Electronics, Coumbia, NC, USA). This consisted of a portable power supply coupled to a UV (320–340 nm) light emitting diode set to the maximum power setting (18 mA). The UV clear optic fibre (0.6 mm diameter) fitted through the urethral cannula (20 gauge) without any leak. The bladder was filled with 0.1 ml sterile PBS to partially distend the bladder and the fibre was advanced so that the tip sat 1–2 mm beyond the end of the cannula. Calculations using the surface area of the fibre tip and the estimated surface area of the bladder (BSA) using the equation BSA=4.83(bladder volume)^2/3^ in cm^2^ (Xiao *et al*, 2003) indicated that a 15 min exposure would be equivalent to 5 kJ m^−2^ UVA delivery.

### Measurement of thymidine and S^4^TdR levels in rat serum

Thiothymidine concentrations in 100 *μ*l samples of rat plasma were determined following acetonitrile protein extraction and comparison with a standard curve of thymidine and S^4^TdR in human plasma. Dried samples were re-suspended in 200 *μ*l of mobile phase (0.05 M NH_3_-Ac pH 5.0). Samples were analysed as described above.

### Histopathology

To confirm that MYU-3L cells retained their carcinoma phenotype, indirect immunoperoxidase staining of paraffin embedded bladder tumour specimens with a pan–cytokeratin cocktail (Abcam, Cambridge, UK) was performed using standard immunohistochemistry protocols. Antigen retrieval was carried out using a citrate buffer and bound antibody was detected using biotin-conjugated rabbit antimouse immunoglobulins and the streptABC kit (DAKO, Ely, Cambridgeshire, UK) and Sigmafast diaminobenzidine tablets (Sigma).

## Results

### Thiothymidine is incorporated into the DNA of bladder cancer cells *in vitro*

**Figure 1:**
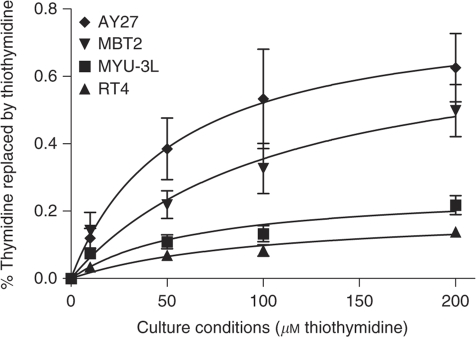
Incorporation of S^4^TdR into cellular DNA. Cell lines were incubated in concentrations of S^4^TdR as indicated on the *x* axis for two cell-doubling times. Following DNA extraction and digestion, nucleosides were analysed using LC/MS/MS to determine the percentage of DNA thymidine replaced by S^4^TdR (*y* axis).

Each of the bladder carcinoma cell lines was assessed for the ability to incorporate S^4^TdR into DNA. Following culture in the presence of 200 *μ*M S^4^TdR for two cell-doubling times, the thiobase replaced ∼ 0.63% of DNA TdR in AY27 cells. Under the same conditions, ∼ 0.50% of DNA TdR was replaced by S^4^TdR in MBT2 cells. The extent of substitution in MYU-3L and RT4 cells was lower, at 0.22 and 0.15%, respectively (Figure 1). These levels of S^4^TdR incorporation are comparable to those reported in human fibroblasts (Massey *et al*, 2001). When AY27 and MYU-3L cells were cultured in a medium with 200 *μ*M thiothymidine, thiothymidine was detectable in DNA extracts after 2 h of incubation, replacing 0.23 and 0.05% for each cell line, respectively.

Raltitrexed increased S^4^TdR incorporation into DNA. Under similar conditions (200 *μ*M, two cell doublings), the addition of IC_5_ and IC_50_ concentrations of raltitrexed increased DNA S^4^TdR incorporation 7- and 11-fold, respectively, in MYU-3L cells. IC_5_ raltitrexed concentrations increased S^4^TdR incorporation 4-, 20- and 4-fold, respectively, in MBT2, RT4 and AY27 cells and these values were even higher at IC_50_ concentrations. These findings are summarised in Table 1.

### Thiothymidine sensitises bladder cancer cell lines to UVA

**Figure 2:**
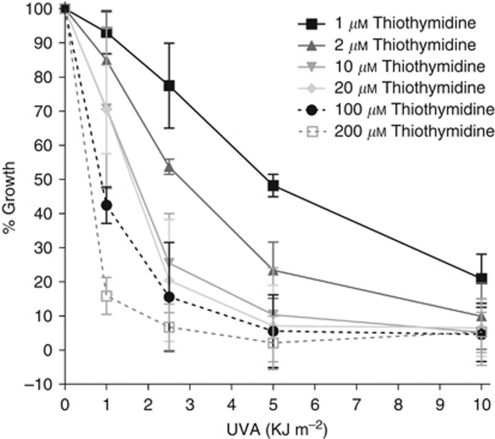
Sensitisation of MYU-3L bladder cancer cells to low doses of UVA. MYU-3L cells were incubated in the presence of S^4^TdR as indicated in the legend for two cell-doubling times. Cells were exposed to UVA (*x* axis) and re-incubated for a further 72 h in fresh medium. Growth was assessed using the SRB assay (*y* axis).

When MYU-3L cells were treated with a range of S^4^TdR concentrations and subsequently exposed to increasing UVA doses, there was a S^4^TdR- and UVA dose-dependent growth inhibitory effect (Figure 2). In control experiments, no growth inhibition was observed in cells treated with S^4^TdR alone up to 300 *μ*M or with UVA at doses less than 50 kJ m^−2^ (data not shown). After 10 and 200 *μ*M S^4^TdR and 1 kJ m^−2^ UVA, growth inhibition was 29 and 75%, respectively, increasing to 89 and 98% growth inhibition at 5 kJ m^−2^. Consistent with their more extensive DNA substitution, a dose of 1 kJ m^−2^ UVA produced 90 and 100% growth inhibition in AY27 cells cultured in 10 and 200 *μ*M S^4^TdR (data not shown).

### UVA sensitisation by S^4^TdR is further augmented by raltitrexed

**Figure 3:**
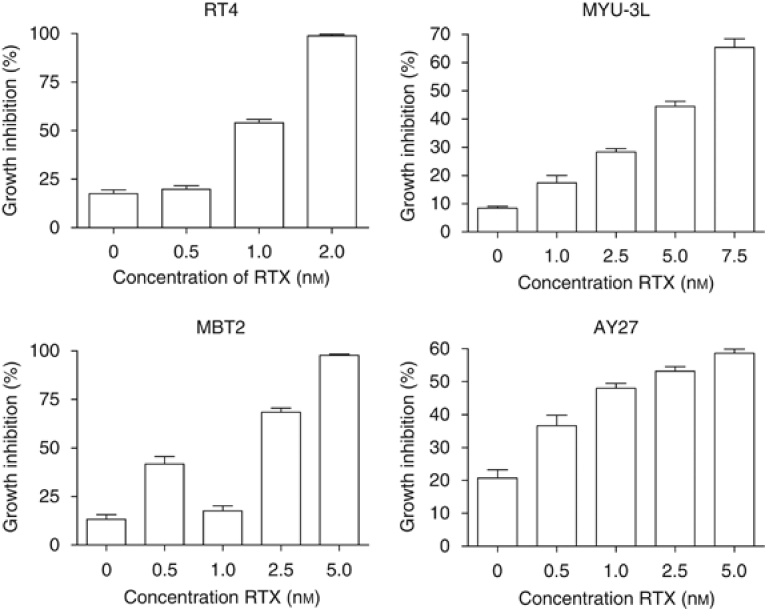
Augmentation of UVA sensitisation using raltitrexed. Cells were cultured for two cell-doubling times with 20 *μ*M S^4^TdR and RTX as indicated. Cells were detached and re-plated in 96-well plates and irradiated under a thin layer of PBS (0.5 kJ m^−2^ UVA for MYU-3L cells and 2.5 kJ m^−2^ for RT4, MBT2 and AY27 cells). After irradiation, 150 *μ*l of fresh growth medium was replaced. Surviving cells were stained and scored after 5 days. All values represent the mean±s.e.m. of 10 replicates.

The enhanced DNA S^4^TdR incorporation after raltitrexed treatment was associated with increased UVA sensitivity. In the absence of raltitrexed, 10 *μ*M S^4^TdR and 0.5 kJ m^−2^ UVA produced a growth inhibition of 8% in MYU-3L cells (Figure 3). When combined with a range of increasing raltitrexed concentrations up to 7.5 nM, the same S^4^TdR concentration and UVA dose resulted in a progressively increasing growth inhibition of up to 65%. Raltitrexed also increased the S^4^TdR/ UVA sensitivity of RT4, MBT2 and AY27 cells. The results for MBT2 and AY27 were comparable to those obtained for MYU-3L cells. Consistent with the more pronounced effect of raltitrexed on DNA S^4^TdR in RT4, the differential sensitivity attributable to raltitrexed was greatest in these cells. (Figure 3). In control experiments, raltitrexed alone did not confer any additional sensitivity to UVA (data not shown).

### Thiothymidine causes DNA crosslinks and apoptosis following UVA exposure

**Figure 4:**
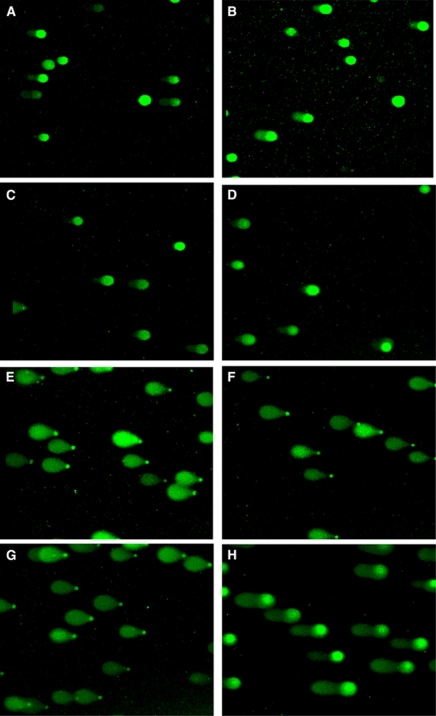
Typical comet images from MYU-3L cells treated with S^4^TdR and UVA. Thiothymidine treatment was for two cell-doubling times at 200 *μ*M and UVA exposure was at 10 kJ m^−2^. Irradiated cells were exposed to 20 Gy X-irradiation. (**A**) Unirradiated control; (**B**) Unirradiated S^4^TdR control; (**C**) Unirradiated UVA control; (**D**) Unirradiated S^4^TdR and UVA; (**E**) Irradiated S^4^TdR control; (**F**) Irradiated UVA control; (**G**) Irradiated control; (**H**) Irradiated S^4^TdR+UVA.

The DNA lesions induced by S^4^TdR/UVA treatment were assessed using the comet assay. The crosslinking of DNA S^4^TdR was measured as a reduction in comet tail moments in X-irradiated cells. Typical comet images are shown in Figure 4. An X-ray dose of 20 Gy induced comets in the nondrug-treated control cells (Figures 4A and G). The length of the comet tail was significantly shorter in X-irradiated cells that had previously been treated with 200 *μ*M S^4^TdR and 10 kJ m^−2^ UVA. The mean reduction in Olive tail moment by S^4^TdR/UVA was 55 and 51% for MYU-3L and AY27 cells, respectively, in three independent experiments. As expected, there was no visible DNA migration in samples that were not X-irradiated. To examine whether formation of DNA–Protein crosslinks by S^4^TdR /UVA contributed to the reduced DNA migration, slides were treated with proteinase K before electrophoresis. Protease digestion caused a 24% increase in tail moment compared with untreated slides (data not shown).

### Thiothymidine/UVA treatment induced apoptosis in the bladder carcinoma cells

**Figure 5:**
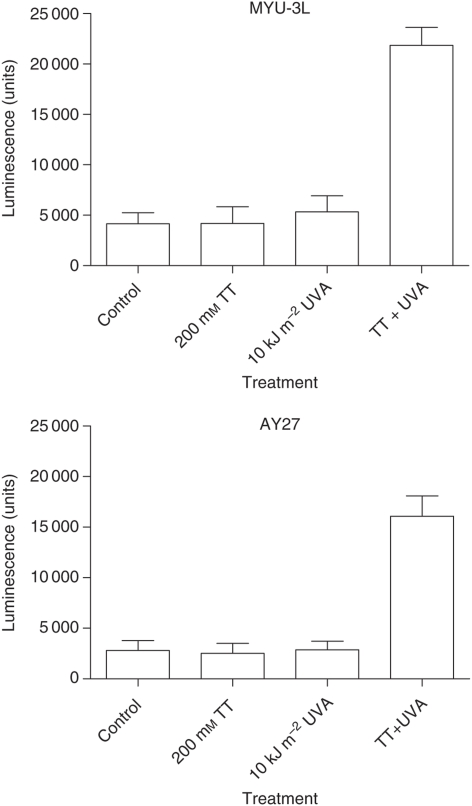
Caspase 3/7 activity following treatment with S^4^TdR and UVA. MYU-3L (above) and AY27 (below) cells were cultured in white walled multi-well plates. Cells were treated with 200 *μ*M S^4^TdR, 10 kJ m^−2^ UVA or a combination of both; four wells were set up for each treatment group. Twenty-four hours after UVA exposure, 50 *μ*l of Caspase-Glo 3/7 reagent was added to each well and incubated for 1 h at room temperature. Luminescence was measured in a plate reading luminometer. The graphs above represent the mean±s.e.m. of three independent experiments each consisting of four replicates.

Twenty-four hours following treatment with 100 *μ*M S^4^TdR and 10 kJ m^−2^ UVA, cells appeared shrunken with condensed nuclei. By 18 h after irradiation, caspase 3 and 7 activity in the culture medium was increased by factors of 5.2 and 5.7 for MYU-3L cells and AY27 cells, respectively, compared with controls (Figure 5). The induction of apoptosis was confirmed by flow cytometry analysis. By 18 h after UV irradiation of cells pretreated with 100 *μ*M S4TdR, annexin binding was detectable in 29 and 26% of MYU-3L and AY27 cells, respectively. The corresponding figures for untreated cells were 5 and 7%.

### Thiothymidine accumulates in DNA of bladder tumours following intravenous or intravesical administration

Bladder tumours were successfully established in F334 Fischer rats by instillation of MYU-3L bladder cancer cell suspensions into preconditioned rats’ bladders. At day 2 after tumour implantation, clumps of cells were visible in the bladder lumen with early adherence to the denuded urothelium. By day 5 post-instillation, stage pT1 bladder tumours were seen with infiltration into the subepithelial connective tissue and tumour depth of <1 mm. Bladders were macroscopically identical to normal rat bladders. Tumours stained positively with a pan–cytokeratin cocktail confirming a carcinoma phenotype. Tumours showed a rapid growth phase from day 6–21 with formation of bulky tumours progressing to muscle invasive and locally advanced disease, but without evidence of metastatic spread. Rat bladders examined histologically between day 5 and day 21 showed tumour development in 45 out of 50 animals inoculated with tumour cells.

Thiothymidine was detectable in bladder tumour DNA following intravenous administration at a dose of 160 mg kg^−1^ (32 mg for a 200 g rat) one week after tumour seeding (Table 2). Tissues were sampled 2 h and 20 h after injection and concurrent blood samples were taken at the time of euthanasia. Specimens of bladder, kidney, liver, skin and eye were obtained from three animals at each time point – one control and two rats in which tumour cells were implanted. Three tissue samples from each organ were analysed per animal. At two hours post-injection, S^4^TdR replaced 0.0018±0.0002% of thymidine in bladder tumour DNA, but was undetectable in DNA from control bladders. By 20 h, substitution in tumour DNA had increased nearly 10-fold to 0.0140±0.0006% and S^4^TdR was present in DNA from control bladders at 0.0055±0.0002%. Thiothymidine was detectable in kidney and liver DNA and the levels of substitution increased between 2 and 20 h (Table 2). Incorporation into skin and eye was much lower (<0.001%). Thiothymidine was cleared quite rapidly from blood; serum concentrations were 27.7±2.0 *μ*M at 2 h and 0.15±0.01 *μ*M at 20 h.

Intravesical instillation of S^4^TdR was also examined. Thiothymidine (0.0040±0.0100%) was present in DNA of bladder tumours immediately after installation for 2 h (16 mg in 0.4 ml sterile water) (Table 2). This was the maximum value observed and the level of substitution decreased at 4 and 20 h post-instillation to 0.0028±0.0010% and 0.0019±0.00003%. In control bladders, S^4^TdR was present at 0.0010% immediately following and at 4 h post-instillation, but was undetectable by 20 h. A similar pattern was observed for other organs, with a maximum S^4^TdR incorporation observed immediately after drug instillation and decreasing with time (Table 2). Plasma S^4^TdR concentration was 0.094 *μ*M immediately after intravesical instillation and 0.052 *μ*M 4 h later. Data were not available for the 20 h time point.

### Thiothymidine and UVA have antitumour activity in an orthotopic model of bladder cancer

The effectiveness of S^4^TdR/UVA as an anticancer treatment was tested in the rat bladder tumour model. The experiment comprised six treatment groups: no-treatment controls, intravenous S^4^TdR only, intravesical S^4^TdR only, UVA only, intravenous S^4^TdR plus UVA and intravesical S^4^TdR plus UVA. All groups contained five rats. MYU-3L tumour cells were instilled in all rat bladders using the intravesical tumour model described above. Five days after implantation, rats in the treatment arm and rats in the S^4^TdR only control group were given S^4^TdR intravenously (160 mg kg^−1^) or intravesically (16 mg in 0.4 ml). After 20 h, the treatment groups and the UVA only control group were anaesthetised and UVA (5 kJ m^−2^) delivered into the bladders via an optical fibre. The control groups were anaesthetised and the UVA probe inserted with no UVA delivery. Fifteen days after the UVA treatment (3 weeks post-tumour implantation), rats were killed. Bladders were removed, weighed to assess tumour bulk, and formalin-fixed for histopathological analysis and tumour staging.

All rats survived in good condition until the end of the study, except for one animal from the UVA only group that displayed significant weight loss and signs of stress. It was killed on day 19 and found to have bilateral pyelonephritis with renal microabscesses and patchy renal infarction. Histological examination revealed a large locally advanced stage pT3 bladder tumour.

**Figure 6:**
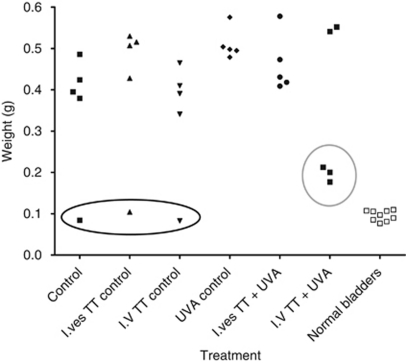
Assessment of tumour burden following S^4^TdR and UVA treatment in rats. Bladders were excised and weighed following thiothymidine and UVA treatment. Tumours failed to establish in three rats (circled black). Responses in the intravenous S^4^TdR and UVA group are circled in grey.

Tumours failed to take in three rats; one each from the no-treatment control arm, the intravesical S^4^TdR only arm and the intravenous S^4^TdR only arm (Figure 6). The bladders from these animals were similar to normal bladders in weight and appeared histologically normal with no evidence of tumour or inflammation. With the exception of the group that received intravenous S^4^TdR and UVA, all other animals developed advanced tumours. Each bladder weighed >0.3 g and histological examination confirmed the presence of advanced carcinoma.

Post-treatment bladder changes were observed in three of the five animals that received intravenous S^4^TdR and UVA (Figure 6). Macroscopic evaluation of the bladders from the three responders revealed some enlargement compared with normal bladders, but significantly less than that of tumour-bearing bladders. Histological examination revealed no evidence of tumour cells. However, there was epithelial proliferation and inflammation with lymphocytes, histiocytes, lymphocytes and eosinophils in addition to haemosiderin deposition. There was no evidence of fibrosis. These findings suggested a proliferative reparatory response with reactive urothelial hyperplasia at the site of inflammation. Importantly, each of the bladders from the responding animals were distinctly different from those in which the tumour failed to implant and from normal bladders that did not show any evidence of inflammation. The bladders of the other two animals in this treatment arm, which were not considered to be treatment responses, contained locally advanced tumours that were macroscopically and histologically identical to the tumours observed in the control groups.

In the group treated with intravesical S^4^TdR and UVA there was no evidence of response to the treatment. There were no observable differences in the macroscopic appearances of the bladders compared with the untreated tumour control groups and microscopic evaluation revealed locally advanced tumours.

## Discussion

Evidence for the potential use of S^4^TdR as a UVA photosensitising agent comes from *in vitro* work with cultured human cells (Massey *et al*, 2001; Reelfs *et al*, 2007). Those initial studies indicated that S^4^TdR is a nontoxic thymidine analog that is incorporated to significant levels into DNA.

Measurements of LC/MS showed that S^4^TdR is incorporated into the DNA of bladder carcinoma cells *in vitro* and can replace up to almost 1% of DNA thymidine without significantly affecting cell growth. Even though all measurements were made after two cell doublings, the extent of S^4^TdR DNA substitution was related to the rate of cell division of each of the cell lines. The AY27 cells had the shortest cell-doubling time and S^4^TdR incorporation in these cells was the greatest. Conversely, RT4 cells grew more slowly and had the lowest rate of S^4^TdR incorporation. These results support the idea that in cells with faster replication, thymidine salvage is important to sustain DNA turnover. Therefore, tumours will incorporate more S^4^TdR than healthy urothelium, as confirmed by the greater incorporation in bladder tumours compared with normal bladder in the orthotopic model.

The presence of S^4^TdR in DNA sensitised each of the cell lines to subsequent UVA exposure. The growth inhibitory effect was dose dependent for both UVA dose and S^4^TdR concentration. Even very low, nontoxic, doses of both S^4^TdR (10 *μ*M) and UVA (5 kJ m^−2^) combined to produce a significant growth inhibition.

The TS inhibitor, raltitrexed, augmented DNA S^4^TdR incorporation in all cell lines and acted as a dose modifier for UVA sensitivity. Pretreatment with IC_5_ concentrations of raltitrexed increased S^4^TdR incorporation by factors of between 4 and 20. The effect of raltitrexed was particularly marked in the cells (RT4 and MYU-3L) in which S^4^TdR incorporation was lowest. These two cell lines had the slowest growth rates and this observation is consistent with the dependence of S^4^TdR incorporation on active thymidine salvage mediated by TK, which is upregulated in rapidly growing cells. A reduced reliance on TS-mediated *de novo* pyrimidine nucleotide synthesis in the rapidly growing MBT2 and AY27 cells results in a less dramatic effect of raltitrexed-mediated TS inhibition on S^4^TdR incorporation. In all cases, the increased incorporation of S^4^TdR was associated with a heightened sensitivity to UVA.

Treatment with S^4^TdR/UVA induces DNA adducts that significantly retard DNA migration in the Comet assay. This effect was partially reversed by proteinase K digestion, indicating that S^4^TdR/UVA causes the formation of DNA–protein crosslinks. These are likely to be difficult lesions for the DNA repair machinery to deal with and they may be a significant contributor to the cell death by apoptosis that was observed as a consequence of S^4^TdR/UVA treatment.

Thiothymidine had no overt toxic effects in rats following either intravesical or intravenous administration of S^4^TdR. Furthermore, there was no significant incorporation of S^4^TdR into DNA of skin and eye, the two tissues most susceptible to photosensitisation. Significant incorporation was noted in liver and kidney, tissues that are not normally exposed to UVA. Significant incorporation into the DNA of bladder tumours was observed following intravenous or intravesical administration of S^4^TdR. The levels of circulating thymidine are particularly high in rats (5–8 *μ*M) compared to humans (0.05 *μ*M) and to that in standard culture medium (0.02 *μ*M). As S^4^TdR has to compete for cellular uptake against this high background level of thymidine, this suggests that S^4^TdR/UVA may be even more effective in humans. Intravenous administration produced a significant level of S^4^TdR incorporation into bladder tumour DNA. The DNA S^4^TdR level at 2 h was somewhat higher following intravesical administration, however, the experimental protocol required that UVA irradiation was performed 20 h after S^4^TdR was administered.

No antitumour response was seen following treatment with intravesical S^4^TdR (16 mg) and UVA (5 kJ m^−2^). The time interval between S^4^TdR administration and UVA delivery is the factor most likely to account for this failure to respond. Based on our DNA S^4^TdR measurements, the optimum time for UVA delivery would have been immediately following S^4^TdR instillation. The constraints of the *in vivo* project licence precluded this treatment option, however.

Three out of the five rats treated with intravenous S^4^TdR and UVA had visible and histological bladder appearances which were markedly different from control rats bladder. Microscopic examination indicated a proliferative repair process with no evidence of tumour cells. This was in contrast to the rats in which tumours failed to take and in which the bladders exhibited normal histology. These histological differences are consistent with an inflammatory process representing repair in the treated animals, and suggesting that their tumours regressed following S^4^TdR and UVA treatment. The timing of UVA administration following intravenous S^4^TdR appeared to be more favourable than for intravesical administration and DNA S^4^TdR was maximal at this time. As S^4^TdR incorporation into DNA in this *in vivo* model was less than that seen to be optimal in *in vitro* experiments, some other mechanism of antitumour effect may be operating. Further investigations would be needed to determine other potential effects of S^4^TdR/UVA, perhaps on tumour vascularisation.

Following the *in vivo* treatments, an active inflammatory process was observed with no evidence of fibrosis. However, fibrosis can be a late complication following intravesical BCG and radiotherapy treatment in humans, which can be functionally disabling. Fibrosis could also be a potential adverse effect of local photodynamic treatment. Urodynamic assessment of rats bladder compliance following photodynamic treatment with 5-aminolavulenic acid (5-ALA) and red light (630 nm) did not previously show any functional changes post-treatment (Xiao *et al*, 2003). There have been reports of severe fibrosis requiring cystectomy in small series of 5-ALA treatment in humans (Walther, 2000).

Thiothymidine is a potent UVA photosensitiser *in vitro*. The *in vivo* work presented here provides the first indication that S^4^TdR is nontoxic in animals and may have potential as an anticancer treatment. It may offer an alternative to existing therapies for hyperproliferative diseases such as cancer, where a photodynamic approach is possible. This study offers a proof of principle that S^4^TdR/UVA can be effective *in vivo*. Further work will be aimed at confirming these promising findings and optimising the timing, dose and route of S^4^TdR administration in an *in vivo* setting as a step towards designing a clinical feasibility study.

## Figures and Tables

**Table 1:** S^4^TdR incorporation into DNA following RTX treatment

		**% S^4^TdR incorporation amount (fold increase)^a^**		
**Cell line**	**Doubling time (h)**	**No RTX**	**IC_5_ RTX**	**IC_50_ RTX**
RT4	35	0.15	2.71 (19.7)	8.16 (59.1)
MYU-3 l	26	0.22	1.55 (7.1)	2.48 (11.4)
MBT2	21	0.50	1.96 (3.9)	2.60 (5.2)
AY27	19	0.63	2.26 (3.6)	4.76 (7.6)

Data from cells cultured in 100 *μ*M S^4^TdR with the stated concentrations of RTX for two cell-doubling times. Fold increases in S^4^TdR incorporation are relative to culture with no RTX.

a The amount of S^4^TdR incorporated is expressed as the percentage of TdR replaced by S^4^TdR. (IC_5_ and IC_50_ concentrations: RT4 2.5 nM, 5.6 nM; MYU-3 L 3.5 nM, 17.5 nM; MBT2 4.0 nM, 8.1 nM; AY27 0.5 nM, 4.0 nM, respectively).

**Table 2:** S^4^TdR incorporation into DNA in different tissues following intravenous (160 mg mk^−1^) or intravesical (16 mg in 4 ml for 2 h) administration.

	**Amount of S^4^TdR incorporated into tissue DNA (% thymidine replaced by S^4^TdR (mean±s.d.))**					
**Route of S^4^TdR administration and time point of tissue extraction**	**Control bladder**	**Bladder tumour**	**Kidney**	**Liver**	**Skin**	**Eye**
*Intravenous S* ^*4*^ *TdR*						
2 h	Undetectable	0.0018 (0.0002)	0.0016 (0.0010)	0.0050 (0.0040)	0.0008 (0.0004)	0.0003 (0.0002)
20 h	0.0055 (0.0002)	0.0140 (0.0006)	0.0040 (0.0030)	0.0200 (0.0100)	0.0005 (0.0004)	Undetectable
						
*Intravesical S* ^*4*^ *TdR*						
Time 0	0.0010 (0.0008)	0.0040 (0.0100)	0.0014 (0.0007)	0.0049 (0.003)	0.0008 (0.0003)	0.0002 (0.0001)
4 h	0.0010 (0.0005)	0.0028 (0.0010)	0.0005 (0.0003)	0.0062 (0.005)	0.0004 (0.0001)	0.0003 (0.0002)
20 h	Undetectable	0.0019 (0.00003)	0.0004 (0.0003)	0.0055 (0.0020)	Undetectable	Undetectable
